# Slow Epidemic of Lymphogranuloma Venereum L2b Strain

**DOI:** 10.3201/eid1111.050821

**Published:** 2005-11

**Authors:** Joke Spaargaren, Julius Schachter, Jeanne Moncada, Henry J.C. de Vries, Han S.A. Fennema, A. Salvador Peña, Roel A. Coutinho, Servaas A. Morré

**Affiliations:** * Municipal Health Service, Amsterdam, the Netherlands; † University of California – San Francisco, San Francisco, California, USA; ‡ Academic Medical Centre, Amsterdam, the Netherlands; § VU Amsterdam Medical Centre, Amsterdam, the Netherlands; ¶ National Institute of Public Health and Environment, Bilthoven, the Netherlands

**Keywords:** lymphogranuloma venereum, chlamydia trachomatis, msm, real-time pcr, dispatch

## Abstract

We traced the *Chlamydia trachomatis* L2b variant in Amsterdam and San Francisco. All recent lymphogranuloma venereum cases in Amsterdam were caused by the L2b variant. This variant was also present in the 1980s in San Francisco. Thus, the current "outbreak" is most likely a slowly evolving epidemic.

Since the end of 2003, an ongoing lymphogranuloma venereum (LGV) proctitis outbreak has been reported in industrialized countries, first in the Netherlands, followed by neighboring European countries and the United Kingdom, and now in the United States and Canada (*1**–**4*). We recently identified a new LGV variant designated L2b (GenBank accession no. AY586530) in all our cases in 2002 and 2003 that suggests this LGV outbreak was new (*5*). Until now, only men who have sex with men (MSM) are affected, and most are HIV co-infected. Although these infections, which can be caused by LGV serovars L1, L2, L2a, and L3, are often characterized by severe inflammatory symptoms, delayed or incorrect diagnosis has increased both the risk for transmission and the development of severe sequelae. Successful treatment of LGV proctitis requires a 3-week course of doxycycline followed by a test of cure, whereas in the case of *Chlamydia trachomatis* proctitis caused by serovars D–K, a 1-week course will suffice.

In a recent article on this LGV outbreak (*3*), 2 issues were stressed: 1) the lack of an easy diagnostic tool and 2) whether lymphogranuloma venereum is a new problem or whether it has been present but undiagnosed. Indeed, among the obstacles to the correct diagnosis of LGV is the lack of a commercially available assay to specifically distinguish between *C. trachomatis* infections caused by LGV serovars and infections caused by less invasive *C. trachomatis* serovars. A definitive diagnosis of LGV is currently made with nucleic acid sequence-based tests, like polymerase chain reaction (PCR)–based restriction fragment length polymorphism (RFLP) analysis, which are only available in a few specialized laboratories. We recently developed a real-time PCR (TaqMan and RotorGene) that can specifically distinguish LGV infections from infections with other *C. trachomatis* serovars, which facilitates diagnosis (*6*).

We used this new diagnostic tool to determine whether the LGV outbreak and its cause are a new phenomenon or whether LGV *C. trachomatis* serovars have been present much longer but have gone undiagnosed. First, we determined if the newly identified Amsterdam L2b variant was already present in the MSM population before 2002 by using stored samples collected from MSM with and without proctitis who sought treatment at the sexually transmitted infections (STI) outpatient clinic in Amsterdam. Second, we performed the same analysis on archived specimens from MSM in San Francisco, California, collected 20–25 years ago.

## The Study

From MSM who attended the Amsterdam Municipal Health Service STI Outpatient Clinic in 2000 and 2001, randomly selected stored specimens of *C. trachomatis* DNA–positive (as assessed by ligase chain reaction, Abbott Laboratories, Chicago, IL, USA) rectal samples were tested for the *C. trachomatis* variant by real-time PCR (*6*). From 2002 to 2005, MSM with symptomatic proctitis (i.e., purulent discharge, rectal ulceration, bleeding, or edematous mucosa) and MSM without symptoms were included.

From the San Francisco region, 51 LGV–positive isolates from symptomatic MSM were analyzed (*7*). The isolates were collected in medical clinics (e.g., ambulatory care, emergency room, screening, acute care) from 1979 to 1985 (Table). LGV was assessed at the time of collection, according to phenotypic properties observed during cell culture. Although the growth characteristics of LGV serovars can be distinguished from serovars D–K, cell culture for *C. trachomatis* is no longer available in most clinical settings.

**Table:** Number of lymphogranuloma venereum (LGV) L2b variants identified in Chlamydia trachomatis DNA–positive rectal swabs in Amsterdam (2000–2005) and San Francisco (1979–1985)

Location	Year*	No. L2b /no. samples
Amsterdam	2000	2/67
	2001	4/28
	2002	40/127
	2003	69/276
	2004	52/297
	2005	26/161
San Francisco	1979	0/1
	1980	0/0
	1981	2/8
	1982	10/29
	1983	5/9
	1984	0/3
	1985	1/1

*In 2002 and 2003, 45 LGV L2b variants of 109 isolates have been described in detail (*5*).

*C. trachomatis* serovar typing was performed as described previously (*5*). Briefly, amplification of the *ompA* gene (1.1 kb) was performed in a nested PCR format. Serovars and variants were initially identified by their RFLP patterns after polyacrylamide gel electrophoresis. The *ompA* nucleotide sequences were subsequently analyzed by automated DNA sequencing on an ABI 310 sequencer (PE Biosystems, Foster City, CA, USA). The sequences obtained from *C. trachomatis*–infected MSM in 2000 and 2001 in Amsterdam and from MSM in San Francisco were compared to the recently identified L2b variant to determine if the strain was present earlier. The Table presents the results of this analysis.

In the Amsterdam *C. trachomatis* DNA–positive rectal samples, LGV strains were detected by real-time PCR in 2 of 67 samples in 2000 and in 4 of 28 samples in 2001. Sequencing showed that in all 6 LGV strain–positive samples, the L2b variant was present. Also in 2002 and 2003, 109 L2b-positive samples of 403 *C. trachomatis* DNA–positive rectal samples were identified, of which 45 were strain L2b, and these have been described in a previous publication (*5*). All 51 San Francisco specimens (from 51 patients) were positive for LGV variants by real-time PCR. By sequencing variable segment 2 of the *ompA* gene (VS-2), we identified 15 as serovar L1, 18 as serovar L2 prototype, and 18 as the L2b variant. We sequenced the complete *ompA* gene of 5 of these 18 L2b variants that originated in San Francisco; all were identical to the recently described L2b variant circulating in Amsterdam. Four nucleotide changes were found when compared to reference serovars L2, L2a, and the variant L2´, including 1 change that encoded the previously undescribed change at amino acid 162, AAT→AGT (*5*).

## Conclusions

The L2b LGV variant identified as the cause of all the LGV proctitis in the recent outbreak among MSM in Amsterdam appears to have been circulating in Amsterdam in 2000. Moreover, we showed that this L2b variant was present in the 1980s in San Francisco with exactly the same mutations in the complete *ompA* gene. However, since we only sequenced the *ompA* gene, and although the sequence was identical in old and new L2b strains, we cannot exclude the possibility that it could involve different strains of *C. trachomatis* that differ in other parts of the genome, although this is unlikely.

Since LGV causes potentially severe infections with possibly irreversible sequelae if adequate treatment is not begun promptly, early and accurate diagnosis is essential. Sequence-based nucleic-acid tests that can discriminate between LGV serovars and less invasive *C. trachomatis* species can help detect cases and prevent further transmission of LGV.

In conclusion, our results suggest that we are dealing with the same LGV variant >25 years later, and the current LGV outbreak in industrialized countries has most likely been a slowly evolving epidemic with an organism that has gone unnoticed in the community for many years and is now being detected by new technologies. The numbers detected in 2005 in Amsterdam suggest that a considerable reservoir exists, which emphasizes the need for ongoing public health awareness.

## References

[1] Nieuwenhuis RF, Ossewaarde JM, Gotz HM, Dees J, Thio HB, Thomeer MG, Resurgence of lymphogranuloma venereum in Western Europe: an outbreak of *Chlamydia trachomatis* serovar 12 proctitis in the Netherlands among men who have sex with men. Clin Infect Dis. 2004;39:996–1003. 10.1086/423966 15472852

[2] French P, Ison CA, Macdonald N. Lymphogranuloma venereum in the United Kingdom. Sex Transm Infect. 2005;81:97–8. 10.1136/sti.2005.015263 15800081 PMC1764657

[3] Blank S, Schillinger JA, Harbatkin D. Lymphogranuloma venereum in the industrialised world. Lancet. 2005;365:1607–8. 10.1016/S0140-6736(05)66490-2 15885284

[4] Kropp RY, Wong T. Emergence of lymphogranuloma venereum in Canada. CMAJ. 2005;172:1674–6. 10.1503/cmaj.050621 15928239 PMC1150253

[5] Spaargaren J, Fennema JSA, Morré SA, de Vries HJC, Coutinho RA. New lymphogranuloma venereum *Chlamydia trachomatis* variant, Amsterdam. Emerg Infect Dis. 2005;11:1090–2. 16022786 10.3201/eid1107.040883 PMC3371808

[6] Morré SA, Spaargaren J, Fennema JSA, de Vries HJC, Coutinho RA, Peña AS. Real-time polymerase chain reaction to diagnose lymphogranuloma venereum. Emerg Infect Dis. 2005;11:1311–2. 16110579 10.3201/eid1108.050535 PMC3320474

[7] Schachter J, Moncada J. Lymphogranuloma venereum: how to turn an endemic disease into an outbreak of a new disease? Start looking. Sex Transm Dis. 2005;32:331–2. 10.1097/01.olq.0000168429.13282.c8 15912077

